# Nocardia infection following ocular surface surgery

**DOI:** 10.1007/s10792-022-02500-5

**Published:** 2022-09-14

**Authors:** Jingting Wang, Xiuhai Lu, Jungang Wang, Shuting Wang, Weiyun Shi, Suxia Li

**Affiliations:** 1 Eye Hospital of Shandong First Medical University (Shandong Eye Hospital), Eye Institute of Shandong First Medical University, No. 372, Jingsi Road, Huaiyin District, Jinan, China; 2 State Key Laboratory Cultivation Base, Shandong Provincial Key Laboratory of Ophthalmology, Shandong First Medical University, No. 372, Jingsi Road, Huaiyin District, Jinan, China; 3 School of Ophthalmology, Shandong First Medical University, No. 372, Jingsi Road, Huaiyin District, Jinan, China

**Keywords:** Nocardia infection, Ocular surface surgery, Amikacin

## Abstract

**Objective:**

To investigate the clinical characteristics and treatment outcomes of Nocardia infection after ocular surface surgery.

**Methods:**

This is a retrospective study. Eight cases of culture-proven Nocardia infection, which developed within 1 month after ocular surface surgery were included. Demographics and clinical history of patients were investigated.

**Results:**

There were 8 eyes (2 left and 6 right) of 8 patients (5 males and 3 females), aged 27–65, with a median age of 52.9 years. Three cases underwent pterygium excision, three were subjected to conjunctival flap covering, and two were treated with lamellar corneal transplantation. The time interval between previous surgery and the onset of symptoms varied from 7 to 28 days (mean = 20.5 ± 7.13 days). All the cases presented grey-white infiltrates at the surgical incision site while appearing with six corneal ulcers and two conjunctival ulcers. Filaments of Nocardia were founded by confocal microscopy in two of the five cases. All responded poorly to medical therapy. Seven of the eight cases were treated with reoperation. Nocardia infection recurred in three cases after reoperation, and one was eviscerated.

**Conclusions:**

Surgical trauma is a risk factor for ocular Nocardia infection. Nocardia infection should be suspected when secondary infection occurs in a surgical incision with an atypical clinical presentation. The use of corticosteroids may influence the efficacy of drugs. Complete removal of lesions may lower the recurrence of Nocardia infection with poor drug treatment effects.

## Introduction

Nocardia is a gram-positive, weakly acid-fast, filamentous bacteria that can cause various ocular infections, such as keratitis, scleritis, and endophthalmitis [1]. Ocular nocardiosis, though has been increasingly reported in recent years, is rarely diagnosed in most parts of the world because of its infrequent occurrence and variable clinical presentation. Nocardia mostly causes opportunistic infections. Trauma [2], topical steroid use [3], ocular surgery, and contact lens wearing are common risk factors associated with ocular nocardiosis [4, 5]. Postoperative infections, especially following surface surgery caused by Nocardia, are rare and reported mostly in single cases [6, 7]. This retrospective study was performed to analyze the clinical characteristics and treatment outcomes of Nocardia infection following ocular surface surgery, contributing to a deeper understanding of the disease and its clinical diagnosis and treatment.

## Subjects and methods

Eight culture-proven Nocardia infection cases within a month after ocular surface surgery were retrospectively reviewed. Detailed clinical history, involving demographics, previous medical history, and previous ophthalmic surgery, was analyzed along with the clinical examination and microbiological findings, as well as medical and surgical treatment. The clinical examination consisted of slit-lamp microscopy, confocal microscopy, anterior segment coherence tomography (OCT), and B-scan ultrasonography.

A metal blade was adopted for specimens that were used for microbiology workup and obtained from the lesion area scrapings. Each sample was smeared on clean glass slides for microscopic examination and then Gram and Calcofluor White (CFW) staining. Kinyoun staining was performed under strong suspicion of Nocardia. The samples were inoculated onto sheep blood agar, chocolate agar, and nutrient broth maintained at 37 °C for 7 days. A positive smear for Nocardia was defined as gram-positive thin branching filaments on Gram stain, a bright blue fluorescence in CFW stain, or Kinyoun staining. The growth of Nocardia species on culture was marked as chalky-white colonies. Nocardia species from clinical isolates were identified using matrix-assisted laser desorption ionization time-of-flight (MALDI-TOF) mass spectrometry. The susceptibility profiles of Nocardia species for 15 antibiotics were determined by the broth microdilution method. Minimal inhibitory concentrations (MIC) interpretive standards for susceptible and resistant strains followed manufacturers and Clinical Laboratory Standards Institute (CLSI, Wayne, PA) guidelines.

Topical broad-spectrum antibiotics treatment was initiated in all patients based on a direct smear examination of the corneal scrapings. Topical 0.3% Gatifloxacin (China Otsuka Pharmaceutical Co., Ltd) combined with tobramycin 3 mg/mL (S.A. Alcon Couvreur N.V.) or 10% cefazolin (Lijian Pharmacy Co., Ltd., China) eye drops were given every 30 min on severe cases. Topical 1% amikacin (Tianfang Pharmaceutical Co., Ltd., China) eye drops were the mainstay of medical therapy once the direct smear examination revealed acid-fast organisms or thin beaded branching filaments. Surgical intervention was considered in cases of poor response to medical therapy. Intact epithelium with no staining on fluorescein application indicated being healed. Recurrence was confirmed if Nocardia reoccurred at the site of the surgical incision for 1 month follow-up time. The final corrected distant vision outcome was collected 2 months after the lesion was healed.

## Results

Eight eyes (2 left and 6 right) of 8 patients (5 males and 3 females), aged 27–65, with a median age of 52.9 years, were diagnosed with Nocardia infection after ocular surface surgery. Two patients had a history of hypertension, and one had a history of Granulomatosis with polyangiitis (GPA). The other five patients were healthy.

**Fig. 1:**
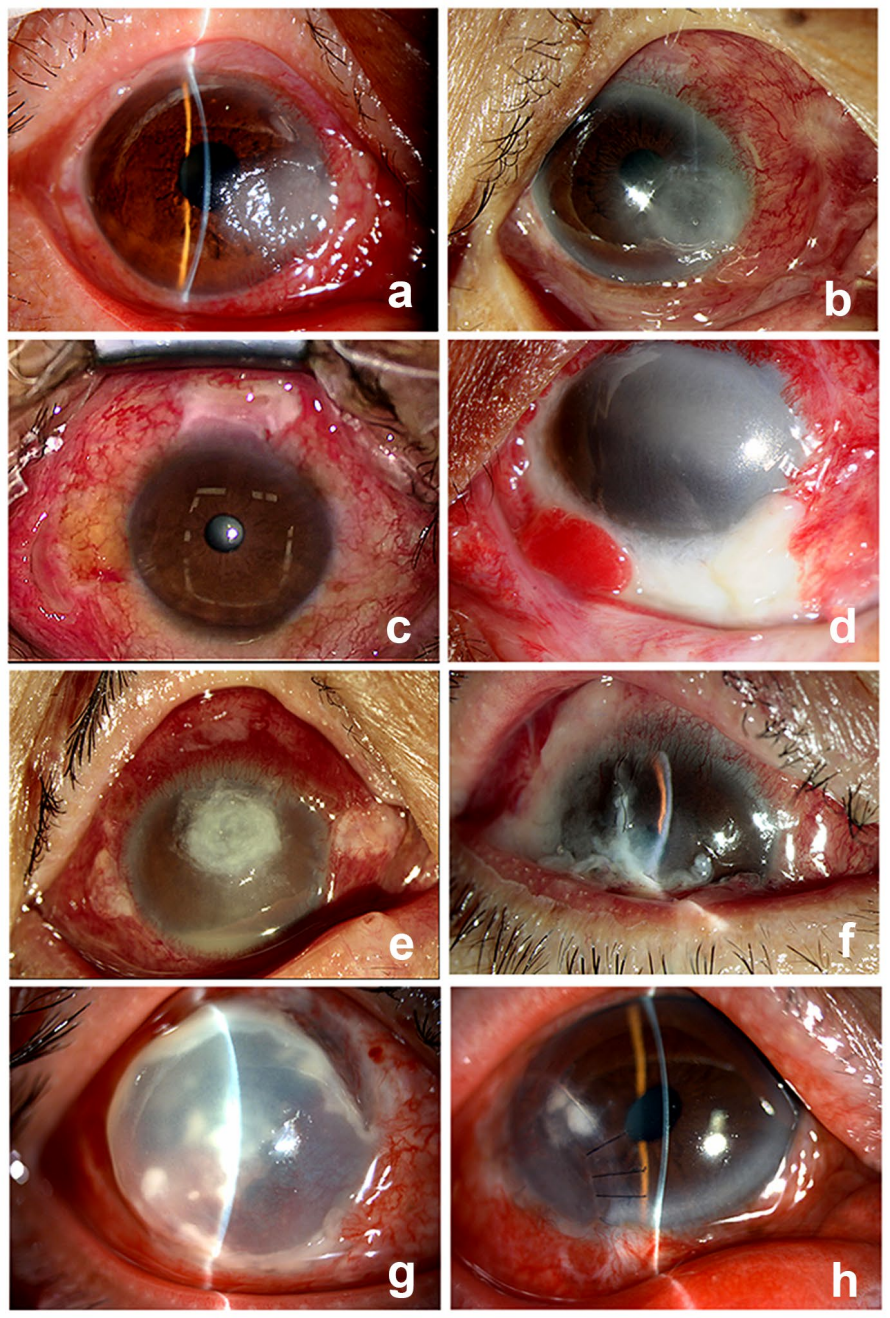
Nocardia infection on slit-lamp examination. Nocardia infection underwent pterygium excision (**a**, **b**, **c**), Nocardia infection followed conjunctival flap covering (**d**, **e**, **f**), Nocardia infection followed lamellar corneal transplantation (**g**, **h**)

All eight patients had undergone ocular surgery within 1 month of the onset of infection at different times. Among them, three had surgery at our hospital, and five cases were referred from other eye centers. None of them was diagnosed with Nocardia infection before the prior surgery. Three patients were treated with pterygium excision (Fig. 1a, b, c), and none of the eyes received antifibrotic (mitomycin C or 5-fluorouracil) adjuvant therapy. Three were subjected to conjunctival flap covering (Fig. 1d, e, f), and two underwent lamellar corneal transplantation (Fig. 1g, h). Fluoroquinolone antibiotics (0.3% Gatifloxacin or 0.5% Levofloxacin eye drops) were used before and after the former surgery. Tobramycin and Dexamethasone eye drops (S.A. Alcon Couvreur N.V.) were taken after the surgery. The time interval between previous surgery and onset of symptoms varied from 7 to 28 days (mean = 20.5 ± 7.13 days).

On slit-lamp examination upon the infection, all the eyes were observed to have infiltrates at the surgical incision site, and the infections were limited to the ocular surface without intraocular involvement. Two cases with corneal ulcers were located at the site of attachment of the head of the pterygium. The infiltrates appeared grey-white with pinhead infiltrates at the edges of the lesion (Fig. 1a, b). There were two conjunctival infections from auto-transplantation of corneal limbus stem cells, and the conjunctival flaps demonstrated grey-white ulcers (Fig. 1c, d). Two cases exhibited grey-white corneal ulcers under the conjunctival flap (Fig. 1e, f). Two cases of corneal graft presented grey-white ulcers (Fig. 1g, h).

**Fig. 2:**
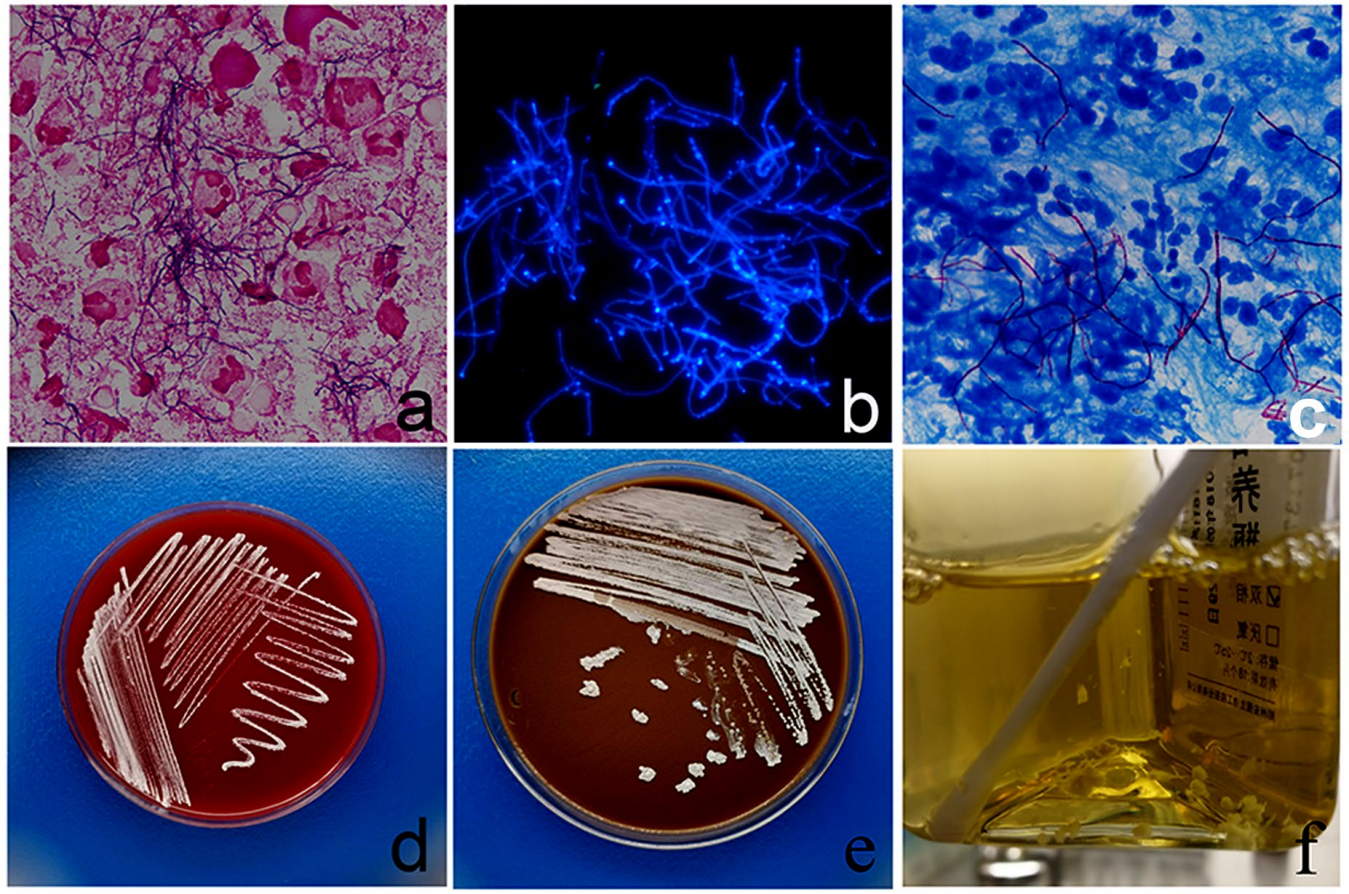
Microbiological examinations of Nocardia infection. Smears on Gram’s stain, CFW, and Kinyoun staining illustrated thin branching filamentous organisms (**a**, **b**, **c**) (1000 × magnification). Blood and chocolate agar plate showed tiny chalky-white colonies of Nocardia at the site of inoculation (**d**, **e**). Nocardia in Nutrient broth (**f**)

Samples from all eight eyes were extracted for microbiological analysis. None of the samples demonstrated bacteria or fungi. Direct smear results and culture of Nocardia species were positive in all cases. Gram’s stain displayed gram-positive, beaded, and branching filaments (Fig. 2a). Bright blue fluorescence branching thin filaments can be observed in CFW stain (Fig. 2b). Kinyoun staining illustrated acid-fast, thin, and branching filamentous organisms (Fig. 2c). Blood and chocolate agar plate demonstrated tiny chalky-white colonies of Nocardia asteroids at the site of inoculation (Fig. 2d, e). Nutrient broth indicated chalky-white colonies of Nocardia clustered at the bottom of the bottle, and the medium was not turbid (Fig. 2f).

**Fig. 3:**
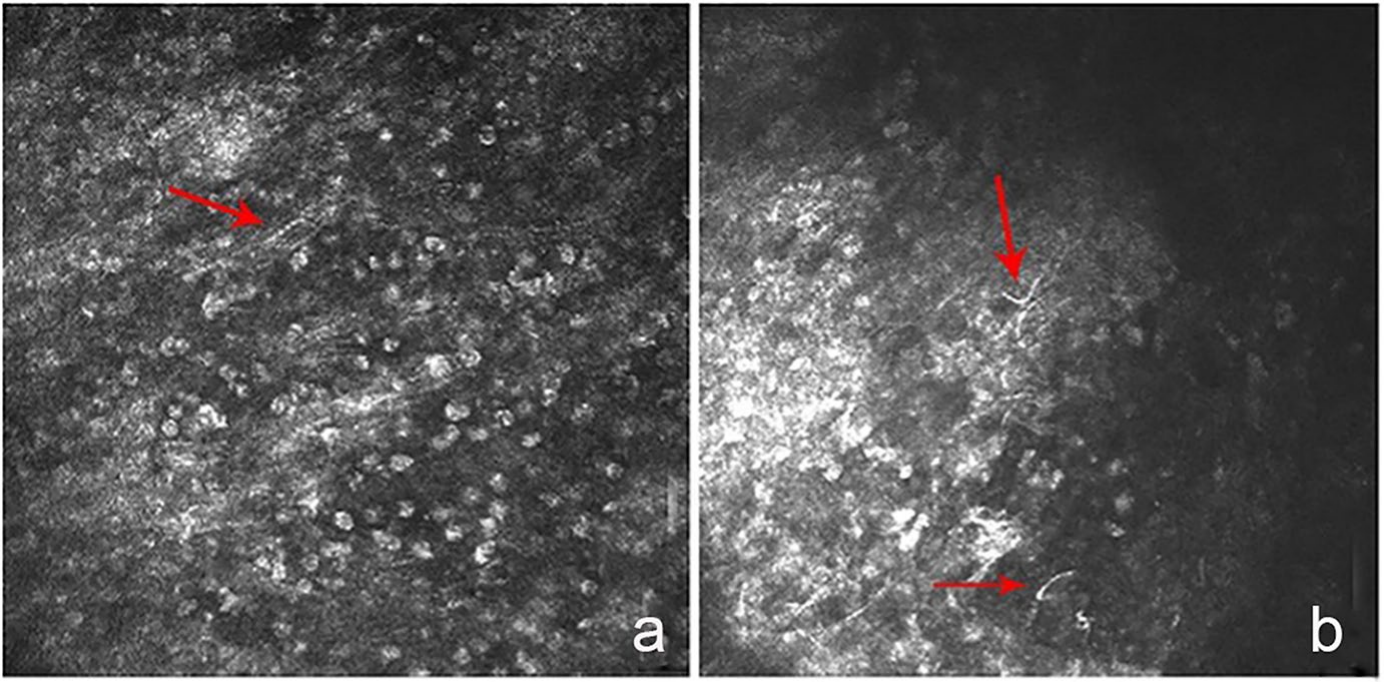
Confocal microscope images of Nocardia. Demonstrate small, slender, beaded filamentous structures in two cases. (**a**, **b**) (800 × magnification) (arrows)

Five of the eight patients obtained confocal microscopy examination, Heidelberg Retina Tomograph III Rostock-cornea-module (HRT III-RCM; Heidelberg Engineering GmbH, Dossenheim, Germany). Filamentous structures suggestive of Nocardia can be detected in two cases. These filamentous structures appeared thin, short, beaded filamentous structures (Fig. 3).

Among these Nocardia isolates, Nocardia abscessus was the most commonly isolated species (62.5%, 5/8), followed by Nocardia asteroids (25%, 2/8) and Nocardia farcinica (12.5%, 1/8). Antibiotic sensitivity testing revealed that all samples were sensitive to Amikacin, linezolid, and trimethoprim-sulfamethoxazole (TMP-SMX). Additionally, it demonstrated low MIC values against different Nocardia species. However, resistance was observed to ciprofloxacin in 6 of 8 isolates (75%), clarithromycin in 4 of 8 isolates (50%), and forβ-lactam antibiotics, including imipenem, cefepime, amoxicillin-clavulanic acid, and ceftriaxone, which all exhibited a poor performance against Nocardia species.

**Fig. 4:**
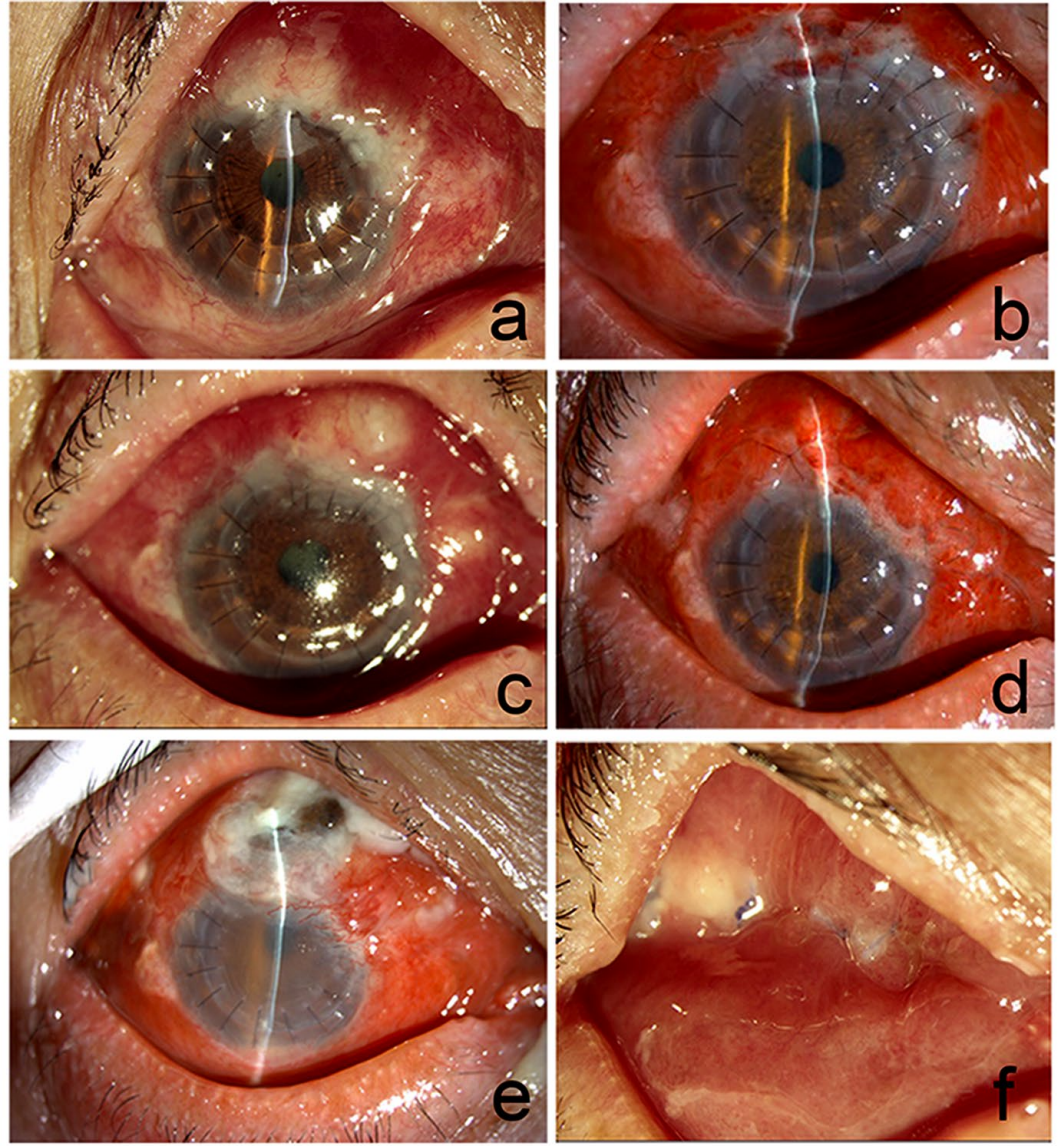
Nocardia recurrence. Nocardia infection on superior conjunctiva 20 days followed PKP (**a**). Infectious lesion resection was performed (**b**). Nocardia infection on superior conjunctiva 15 days following resection (**c**). Resection was performed again (**d**). Scleritis and endophthalmitis recurred (**e**). Nocardia infection after eviscerated (**f**)

However, all the patients responded poorly to drug treatment. One patient with GPA history and Nocardia infection after full lamellar keratoplasty gave up treatment. Seven of the eight cases required some form of surgical intervention, four obtained keratoplasty, and three were treated by infectious lesion resection. Nocardia recurred in three of the seven reoperated patients (42.8%). Corneal ulcers were cured by eye drops (1% Amikacin) in two of them. One of the patients (Fig. 1e) with conjunctival Nocardia infection 20 days following penetrating keratoplasty (PKP) (Fig. 4a) responded poorly to Amikacin despite the positive antibiotic sensitivity testing. Infectious lesion resection was performed twice (Fig. 4b, c, d). However, infectious scleritis and endophthalmitis occurred, and the abscess recurred at the lesion and even was eviscerated (Fig. 2e, f).

## Discussions

Nocardia species are ubiquitous and can be discovered in water, soil, dust, and decaying vegetation worldwide [8]. Nocardia can be found as a saprophyte on the skin and upper respiratory tract, while it does not present as normal flora within the eye [9]. Corneal and conjunctival tissue damage is the pathological foundation of the Nocardia ocular infection. Surgical trauma is one of the most common risk factors for Nocardia ocular infection. Former reports of Nocardia infection after surgeries mostly involved single cases, such as Femtosecond Laser-Assisted Lamellar Keratectomy and cataract surgery [5, 10–12]. This paper reported eight Nocardia infection cases following ocular surface surgery: three patients with pterygium excision, three patients undergoing conjunctival flap covering, and two patients subjected to lamellar corneal transplantation. They all had a large area of conjunctiva cut open during the operation. As a result, the conjunctiva’s integrity was destroyed, and the conditioned pathogenic bacteria were exposed to the conjunctiva wound, leading to a higher chance of secondary infection. Moreover, all the lesions were infiltrated at the site of the surgical incision. Garg [6] and Javadi [13] reported three patients (four eyes) with the outbreak of Nocardia keratitis after corneal refractive surgery. They suspected that the most probable cause of the outbreak was non-standard sterilization and non-aseptic operation. In this study, operations of all the patients were performed by different surgeons at different times. There was no cluster infection.

Generally, Nocardia infection does not occur in clinical practice, with a global prevalence of below 2% [14]. Being mistaken for fungal or viral keratitis leads to delayed treatment and an increased risk of permanent visual impairment. This delayed diagnosis may be induced by a lack of familiarity with this uncommon pathogen and its variable presentation. The classic description of Nocardia keratitis was a yellow-white discrete pinhead-sized appearance in the infiltrated area, forming a characteristic wreath pattern [15]. None of the patients was suspected of Nocardia after the initial clinical examination. Hence, atypical clinical appearance may be confused with that of herpes simplex keratitis or fungal keratitis. Delay in the identification sometimes results in injudicious usage of topical corticosteroids, which may exacerbate the infection. Topical corticosteroid eye drops are used in all patients in our series after their initial surgery. One patient with a history of GPA had used oral corticosteroids previously for many years. Case reports have suggested that topical corticosteroids may result in recurrence of the infection and larger infiltrate/scar sizes [16, 17]. Our study unveiled that the use of adjunctive corticosteroids was associated with an atypical clinical appearance and poor response to the sensitive drugs.

Amikacin has been effective and is considered the first-line drug of choice in Nocardia infection [18, 19].

Nevertheless, its susceptibility may differ with geography or diverse isolates. Susceptibility testing is crucial to determine the most effective drugs for ocular Nocardia infections. All of the Nocardia strains were susceptible to amikacin in this current study, consistent with the largest-sample-size surveillance study on Nocardia strains and nocardiosis throughout China [20]. However, all the patients responded poorly to amikacin in this current study. Seven of the eight cases required some form of reoperation. The use of corticosteroids contributed to this phenomenon. Four recurrences occurred in one patient after conjunctival flap covering. Sensitive antibiotics Amikacin and infectious lesion resection responded poorly. Moreover, recurrence appeared and even was eviscerated. This might be in that the rollback conjunctival flap was not checked and excised during the reoperation procedure. Hidden Nocardia or Nocardial scleritis may exist under the conjunctiva. This case suggested that early surgical intervention must remove the infectious lesion completely to reduce the recurrence. Unfortunately, whether it was usefully treated with sub-tenon or intravitreal injections of amikacin in the early phase was not observed in our study, and other sensitive antibiotics such as linezolid and TMP-SMX are inapplicable due to the restrictions in the current hospital.

The typical first-line prophylactic preoperative application of ocular antibiotics, such as fluoroquinolones, was frequently reported to resist this pathogen [21]. In the current study, resistance was observed to fluoroquinolones in 6 of 8 isolates (75%). Nocardia infections should be suspected when secondary infection occurs in a surgical incision with an atypical clinical presentation.

Previous reports have verified that a confocal microscope can visualize Nocardia, revealed as multiple, thin (< 1.5 μm), short, and beaded filamentous structures that demonstrated right-angled branching ^[4, 21]^. Described filamentous structures of Nocardia were detected by confocal microscopy in two of our cases. The manufacturer quotes the minimum optical transverse resolution of 1 μm with the HRT III-RCM. Nocardia thinner than 1 μm may be difficult to detect owing to a limitation of the resolution. The sensitivity and positive predictive values in diagnosing Nocardia keratitis may increase, especially in cases where scarp cannot be achieved, with the increasing use of the new-generation confocal microscopy that has greater magnification (800 ×) and clearer images than that of earlier generation microscopy (380 ×) as a noninvasive diagnostic tool.

There are several limitations to this study. In the current study, only eight samples were isolated, resulting in a small number of each Nocardia species. Additionally, a standardized treatment protocol is deficient. However, the current series of Nocardia infections following ocular surface surgery was first reported, and some essential clinical and microbiological information was revealed in the management of these patients.

## Conclusions

Surgical trauma is a risk factor for ocular Nocardia infection. Nocardia infection should be suspected when secondary infection occurs in a surgical incision with an atypical clinical presentation. The use of corticosteroids may influence the efficacy of drugs. Complete removal of lesions may reduce the recurrence of Nocardia infection with poor drug treatment effects.
